# Veterinary Medical Association of New York County

**Published:** 1896-11

**Authors:** Robert W. Ellis

**Affiliations:** Secretary


Secretary.

### VETERINARY MEDICAL ASSOCIATION OF NEW YORK COUNTY.

THE regular monthly meeting was called to order at 8.30 P.M., October 7th, by the President, Dr. Huidekoper, at the Academy of Medicine. The following members responded to the roll-call: Drs. Delaney, Dair, Ellis, Giffen, Gill, Huidekoper, Hanson, Loomes, Machan, MacKellar, Neher, Robertson, and Ryder (13). The minutes of the previous meeting were read and approved.

Moved and seconded that the report of the Board of Censors (Dr. H. D. Gill, chairman) be voted on in sections; carried.

1. Johnson's case. 2. Ferster's case. 3. Glover's case. 4. Recommendations to be presented by the chair to Judiciary Committee.

The charge against Dr. S. K. Johnson, for breach of Article VII. of Code of Ethics, was referred back to the Association for the reason that the Board of Censors had no jurisdiction, as a motion relative to the case had been passed at a meeting held April 7, 1896, which was as follows: "Resolved that the Board of Censors recommend to the Association that if the said Dr. S. K. Johnson does not show satisfactory evidence of having resigned and severed all connection, directly or indirectly, with the said insurance company on or before October 15, 1896, he be expelled as a member of this Association." Moved and seconded that the report be accepted; carried.

The acceptance of Dr. J. H. Ferster's resignation was at the last meeting laid on the table. The Board of Censors finds that Dr. Ferster, at the date of his resignation, was in good standing; therefore recommends the acceptance of the same. Moved and seconded that the report be accepted; carried.

The charges against Dr. H. Clay Glover were for a breach of Article VI. of Code of Ethics and the improper and illegal use of the title D.V.S., and he was recommended to the Association for expulsion, and they also suggest that the attention of our Judiciary Committee be called to the latter fact. Moved and seconded that the report be accepted; carried.

Information having come to the Board of Censors that meat-inspectors employed by the New York City Health Department and the Bureau of Animal Industry were not veterinarians, and consequently illegally practising, the matter was referred to the Judiciary Committee for immediate action.

Dr. W. Herbert Lowe, of New Jersey, read a very excellent and carefully prepared paper on "Heredity." The discussion was opened by Dr. Hanson, and followed by Drs. Gill, Neher, and others. Moved by Dr. Hanson and seconded by Dr. Robertson that a vote of thanks be extended to Dr. Lowe for his paper and interest shown in the Association; carried.

The Board of Health Committee (Dr. Robertson, chairman) reported progress. The committee was then directed by the chair to continue for another month, and report in writing at the next meeting.

The following gentlemen presented applications for membership in the Association: F. E. Winslow, of Flushing, L. I., and J. W. H. Wright, of Long Island City.

Moved and seconded that a vote of thanks be extended to visiting members for their courtesy in accepting the Association's invitation to be present at the meeting; carried.

Moved and seconded that the meeting adjourn; carried.

ROBERT W. ELLIS, D.V.S.,
Secretary.

